# Laparoscopic uterovaginal prolapse surgery in the elderly: feasibility and outcomes

**DOI:** 10.1186/s10397-017-1000-x

**Published:** 2017-04-11

**Authors:** Samuel W. King, Helen Jefferis, Simon Jackson, Alexander G. Marfin, Natalia Price

**Affiliations:** 1 Department of Urogynaecology, Oxford University Hospitals, Oxford, UK; 2 Oxford Medical School, John Radcliffe Hospital, University of Oxford, Oxford, UK; 3 John Radcliffe Hospital, Oxford University Hospitals NHS Foundation Trust, Oxford, UK; 4 Harrogate District Hospital, Lancaster Park Rd, Harrogate, UK

**Keywords:** Elderly, Hysteropexy, Laparoscopic, Prolapse, Sacrocolpopexy

## Abstract

**Background:**

Uterovaginal prolapse in very elderly women is a growing problem due to increased life expectancy. Surgeons and anaesthetists may be wary of performing quality of life surgery on this higher risk group. Where surgery is undertaken, it is commonly performed vaginally; there is a perception that this is better tolerated than abdominal surgery.

Little data is published about laparoscopic prolapse surgery tolerability in this population, and laparoscopic surgery is perceived within the urogynaecological community as complex and lengthy and hence inherently unsuitable for the very elderly.

In Oxford, UK, laparoscopic abdominal surgical techniques are routinely employed for urogynaecological reconstructive surgery. The authors offer abdominal laparoscopic prolapse surgery to patients suitable for general anaesthesia with apical vaginal prolapse, irrespective of age. We here report outcomes in this elderly patient cohort and hypothesise these to be acceptable.

This is a retrospective case note review of all patients aged 79 years old and above undergoing laparoscopic prolapse surgery (hysteropexy or sacrocolpopexy) in two centres in Oxford, UK, over a 5-year period (*n* = 55). Data were collected on length of surgery, length of stay, intraoperative complications, early and late post-operative complications and surgical outcome.

**Results:**

Mean age was 82.6 years (range 79–96). There were no deaths. Minor post-operative complications such as UTI and constipation were frequent, but there were no serious (Clavien-Dindo grade III or above) complications; 80% achieved objective good anatomical outcome.

**Conclusions:**

Laparoscopic prolapse surgery appears well tolerated in the elderly with low operative morbidity and mortality.

## Background

Older women are disproportionately affected by pelvic prolapse compared with their younger counterparts. Those in their eighth decade generate ten times as many consultation hours regarding pelvic prolapse as those in their fourth [1]. “Elderly” is frequently defined in the literature as over 65 years of age, and the safety and effectiveness of vaginal prolapse surgery has been analysed in this group previously. Indeed, some papers define “elderly” at an even younger age [2]. However, few clinicians would now regard a 66-year-old as elderly. For the purposes of this study, “elderly” is defined as those patients in their 80th year and beyond. This age cut-off is arbitrary, and clearly, “biological age”, reflecting concomitant disease, is of more relevance for many than chronological age. However, whilst many surgeons have few qualms operating on patients in their 70s, those over 80 are often viewed with more concern.

The population of people age over 75 in the UK is expected to double and that of those over 85 nearly treble in the next 30 years [3]. With a higher proportion of elderly people being women [4], vaginal prolapse is a significant and growing burden on health services. Prolapse significantly impacts quality of life in this population, causing discomfort, urinary symptoms, and psychosexual issues. However, surgeons and anaesthetists may be wary of operating on this age group as surgery is life-enhancing rather than life-saving. Traditionally, vaginal repair surgery performed includes vaginal hysterectomy, anterior/posterior colporrhaphy and sacrospinous fixation. All of these carry a significant morbidity, such as secondary haemorrhage, infection and exacerbation of pre-existing medical problems. These risks increase in the very elderly. We hypothesise that reconstructive laparoscopic surgery, with low infection risk and little risk of significant blood loss, is an acceptable alternative option for this age group.

Data were collected for patients aged 79 and above who underwent laparoscopic hysteropexy or sacrocolpopexy, with or without additional procedures, as operated on by or under direct supervision of the two consultant gynaecological surgeon authors across two centres in Oxford. The primary aim was to evaluate morbidity and mortality in this cohort.

## Methods

Approval was granted by the regional audit committee to retrospectively analyse the records of patients aged 79 years of age and over undergoing laparoscopic sacrocolpopexy or hysteropexy over a period of 5 years. Ethics approval was not required as this was a retrospective case note review.

The inclusion criteria were as follows: patients of 79 years and older at the time of procedure, undergoing either laparoscopic sacrocolpopexy or hysteropexy, and with or without additional procedures. When seen in clinic, patients with symptomatic vaginal apical prolapse are counselled regarding both conservative and surgical management options. If surgery is chosen, and they are suitable for general anaesthesia, both vaginal and laparoscopic surgery is discussed. Our preference is laparoscopic abdominal surgery as our experience suggests this is well tolerated and effective. However, if not suitable for general anaesthesia, then abdominal laparoscopic surgery is contraindicated.

Notes were reviewed retrospectively for all patients fitting the inclusion criteria over a period of 5 years (2010–2015). Both paper notes and electronic records were reviewed, including pre-operative assessments, intraoperative notes, anaesthetic notes, post-operative in-patient notes, discharge summaries and follow-up clinic letters.

Prolapse was objectively assessed in clinic pre-operatively by measuring and graded using the pelvic organ prolapse quantification (POP-Q). American Society of Anaesthesiologists (ASA) grades were calculated by a consultant anaesthetist and used as a proxy for pre-operative morbidity of patients. ASA grade is scored from 1 to 6, where 1 is a healthy person, 2 corresponds to mild systemic disease, and 3 to severe systemic disease. None of the included patients had a score of 4 (severe disease which is a constant threat to life) or above.

Surgical technique was as previously described [5, 6]. Antibiotic prophylaxis was used in all cases as per local trust surgical site infection guidelines. Venous thromboembolism risk assessment was performed for all cases, and all were prescribed post-operative low molecular weight heparin until discharge.

Data collected included length of surgery, intraoperative complications, early post-operative complications (defined as from end of surgery to discharge from hospital) and late post-operative complications (defined as from discharge to outpatient follow-up). Complications were classified using the Clavien-Dindo surgical complication classification system [7].

## Results

Fifty-five women aged 79 and over were operated on during the 5-year study period. They had a mean age of 83 (range, 79–96 years) on the day of procedure. One woman underwent laparoscopic hysteropexy twice over the time period, and so this was counted as two procedures, resulting in 56 procedures in total; 26 were laparoscopic hysteropexy, and 30 were laparoscopic sacrocolpopexy. During the same time period, only nine patients aged 79 and over underwent vaginal procedures for apical prolapse (vaginal hysterectomy, sacrospinous fixation or both).

There were seven (12.5%) patients with an ASA grade of 1, 40 (71.4%) with a grade of 2 and eight (14.3%) with a grade of 3. One patient did not have an ASA grade or comorbidities reported in their notes. There were no patients with POP-Q stage of 1, eight (14.3%) with a grade of 2, 26 (46.4%) with a stage of 3 and 19 (34%) with a stage of 4.

Twenty-eight patients (50%) had concomitant additional procedures which included anterior and/or posterior colporrhaphy and oophorectomy. For those without additional procedures, mean duration of laparoscopic surgery was 76 min (range, 35–145 min), whilst mean duration of surgery with additional procedures was 82 min (range, 35–205). Three of the procedures were performed by a urogynaecology subspecialty registrar under the supervision of a consultant urogynaecologist, whilst the rest were performed by one of the two consultant co-authors (NP and SJ).

One patient sustained a bladder injury intraoperatively which was repaired during the procedure, and did not affect the operative outcome. There were no other intraoperative complications. Blood loss was not formally measured but estimated at less than 100 ml in all cases; no patient had clinical indication for a post-operative full blood count measurement, and no patient required a blood transfusion.

In the early post-operative period, there were ten grade I and nine grade II complications. In the late post-operative period, there were nine grade I and four grade II complications. This is summarised in Table 1. There were no grade III or higher complications.

**Table 1:** Frequency of early and late post-operative complications

Clavien-Dindo complications	Early (theatre to discharge)	Late (discharge to follow-up)
Grade I		
Constipation	6	7
Diarrhoea	1	0
Voiding difficulty	1	2
Faecal incontinence	1	0
Grade II		
UTI	3	3
Hypertension	2	0
Vaginal infection	1	1
Fall	1	0
Pneumonia	1	0
Altered ECG	1	0

Clavien-Dindo scoring system used: grade I is any deviation from the normal post-operative course without the need for pharmacological treatment or surgical, endoscopic and radiological interventions. Grade II are those requiring pharmacological treatment with drug other than such allowed for grade I complications. No higher category complications occurred

The mean length of stay was 2.3 days (range, 1–15 days). With the exception of one particularly complex patient with a large number of comorbidities and very large prolapse protruding 20 cm beyond the introitus (POP-Q stage IV), mean length of stay was 2.0 days (range, 1–5 days). There was a weak positive correlation between ASA grade and length of stay (*r* = 0.35) and none between age and length of stay (*r* = 0.08).

Six patients did not attend follow-up clinic. All six were still living at time of data collection according to their electronic patient records, and had not been admitted or referred back by their general practitioner for complications relating to their procedure, or attended hospital for complications related to their procedure. The rest attended with follow-up taking place at a mean period of 73 days post-operatively (range, 20–186 days). Forty-five (80%) patients had objective evidence of good anatomical results with no remaining or recurring prolapse. Five (9%) had prolapse requiring further surgery whilst six (11%) had minor residual prolapse with minimal symptoms.

## Discussion

Stepp et al. [8] investigated the outcomes of urogynaecological surgery in 267 women over 75 years old. They found that the overall perioperative morbidity rate in elderly women who underwent urogynaecological surgery was low. However, the vast majority of procedures were solely via a vaginal approach, and those approached laparoscopically were not separated out on analysis.

Friedman et al. [9] compared 120 women over the age of 79 undergoing non-laparoscopic gynaecological surgery with 1497 younger women. Although length of stay for the elderly was slightly increased, mortality and complication rates were comparable to that of younger patients. They concluded that age need not be the sole determinant in the decision to undergo major elective gynaecological surgery.

Laparoscopic hysteropexy and sacrocolpopexy are reported as safe and effective for the treatment of prolapse in younger populations [10, 11]. These procedures are, however, regarded by many as complex major surgery that should consequently be avoided in the elderly. However, laparoscopic surgery has been evaluated in the elderly population in general surgery in cholecystectomy and ventral rectopexy, with the conclusion that it is well tolerated [12, 13]. Little has been published specific to laparoscopic prolapse surgery in older patients, and that which has been published has mixed conclusions. Elneil et al. described a series of 19 patients aged over 76 undergoing laparoscopic prolapse surgery and concluded it offered a good alternative to the vaginal approach [14]. Turner et al. however compared patients over and under the age of 65 undergoing laparoscopic sacrocolpopexy and found that the older group had higher rates of complications, concluding that age was a significant predictor of post-operative morbidity [15]. Our own data suggest age does not preclude surgery, and specifically laparoscopic abdominal surgery is not contraindicated. Indeed, during this study period, the majority of our elderly patients were treated with this approach; only nine other over 79 years old were treated surgically within our unit (vaginal hysterectomy or sacrospinous fixation) during the study period.

There was one intraoperative complication; this was not age related: a bladder injury was treated intraoperatively without impacting on the planned procedure, and she made an uneventful post-surgical recovery. No patient experienced a grade III or higher post-operative complication. Whilst grade I and II complications were relatively common, none of these resulted in major harm or long-term detriment to the patient. The most frequent complications, both early and late, were constipation and urinary tract infection with cumulative rates of 8.9 and 19.6%, respectively. These are both common complications of prolapse surgery, as shown in a younger cohort undergoing hysteropexy where rates were 12.9% for constipation and 2.9% for UTI are reported [10]. Whilst the incidences of both of these complications were increased in our more elderly cohort, elderly patients in general have a greater frequency of constipation and UTI. There was no statistically significant difference in the rate of Clavien-Dindo grade II complications in the early post-operative period in our patients compared with that in laparoscopic rectopexies in patients over 80 years of age (*p* = 0.98) [13]. The overall objective cure rate of this surgery was 80%. In a previous study of laparoscopic sacrocolpopexy in younger women by our group, the objective cure rate was 88% [5], perhaps suggesting a reduction in the quality of connective tissue in older women.

Urogynaecologists have traditionally favoured vaginal surgery over abdominal, because the vaginal approach has been considered less invasive, with shorter post-operative recovery, and lower morbidity as compared with a laparotomy. However, this is now changing as minimally invasive laparoscopic techniques allow an abdominal approach without laparotomy. There is a perception that laparoscopic surgery is not suitable for the elderly, because general anaesthesia is mandatory, and the surgery can be prolonged and complex. Our data indicate this is not valid.

The results from our series indicate a laparoscopic abdominal approach is safe in this age group. However, the surgery was performed in a centre with a high laparoscopic urogynaecology workload, and the surgeons are very familiar with laparoscopic techniques and operate relatively swiftly. Sacrocolpopexy operation duration varies considerably in differing studies [16, 17]; whether surgery in the elderly would be well tolerated if operations took 2–3 h to perform is debatable and has not been tested by our study.

A limitation of this study is a relatively short follow-up time post-operatively; it would be desirable to assess satisfaction with outcome and incidence of surgery-associated complications over a longer time period. However, the purpose of this study was to investigate safety in the elderly; we have previously published on laparoscopic efficacy [10]. Perioperative and post-operative complications associated with elderly patients would be expected to occur within 6 weeks of surgery, and our study captures this data. A further limitation is the use of ASA score, as this does not fully capture the frailty of elderly patients. However, our literature search has confirmed that there is no specific perioperative risk scoring system for the elderly, and so this widely used scoring system was used as the best approximation.

## Conclusions

This series suggests that contrary to popular belief, laparoscopic prolapse surgery is well tolerated in an ageing population. Laparoscopic prolapse surgery in experienced hands has comparable operative times to open surgery, and the avoidance of laparotomy reduces the risk of infection, haematoma, wound dehiscence and hernia formation, none of which were seen in this study. It allows the advantages of abdominal mesh to be conferred to the elderly. In other studies, this has reduced recurrence rates compared with non-mesh surgery and extrusion rates when compared with vaginal mesh surgery. We conclude that laparoscopic prolapse surgery is an acceptable approach for the very elderly.

## Abbreviations

ASA: American Society of Anaesthesiologists
POP-Q: Pelvic organ prolapse quantification
